# Risk factors for severe cases of COVID-19: a retrospective cohort study

**DOI:** 10.18632/aging.103803

**Published:** 2020-08-15

**Authors:** Feng He, Qingqing Luo, Ming Lei, Lixin Fan, Xinning Shao, Guanglie Huang, Jun Zeng, Ziwen Zhao, Shuguang Qin, Zhi Yang, Na Yu, Liuping Yang, Jie Cao

**Affiliations:** 1 Guangzhou First People’s Hospital, The Second Affiliated Hospital of South China University of Technology, Guangzhou, China; 2 Guangzhou Eighth People’s Hospital, Guangzhou Medical University, Guangzhou, China

**Keywords:** COVID-19, severe cases, risk factors, clinical characteristics

## Abstract

Background: SARS-CoV-2 has raged around the world since March, 2020. We aim to describe the clinical characteristics and risk factors of severe patients with COVID-19 in Guangzhou.

Results: The severity and mortality of COVID-19 was 10.4% and 0.3% respectively. And each 1-year increase in age (OR, 1.057; 95% CI, 1.018-1.098; P=0.004), Wuhan exposure history greater than 2 weeks (OR, 2.765; 95% CI, 1.040-7.355; P=0.042), diarrhea (OR, 24.349; 95% CI, 3.580-165.609; P=0.001), chronic kidney disease (OR, 6.966; 95% CI, 1.310-37.058; P = 0.023), myoglobin higher than 106 μg/L (OR, 8.910; 95% CI, 1.225-64.816; P=0.031), white blood cell higher than 10×10^9^/L (OR, 5.776; 95% CI, 1.052-31.722; P=0.044), and C-reactive protein higher than 10 mg/L (OR, 5.362; 95% CI, 1.631-17.626; P=0.006) were risk factors for severe cases.

Conclusion: Older age, Wuhan exposure history, diarrhea, chronic kidney disease, elevated myoglobin, elevated white blood cell and C-reactive protein were independent risk factors for severe patients with COVID-19 in Guangzhou.

Methods: We included 288 adult patients with COVID-19 and compared the data between severe and non-severe group. We used univariate and multivariate logistic regression methods to explore risk factors of severe cases.

## INTRODUCTION

In December 2019, a large-scale infectious pneumonia of unknown origin broke out in Wuhan, China. Chinese scientists isolated a new coronavirus, SARS-CoV-2, causing the pneumonia on Jan 7, 2020 [1, 2]. And WHO named it Coronavirus Disease 2019 (COVID-19) in February 2020 [3]. Since March, justifying the previous data model [4], COVID-19 has raged across world. Up to Jun 3, 2020, there have been more than 6.4 million diagnosed cases in more than 200 countries, with a mortality rate of about 6% [5].

The clinical manifestations of COVID-19 range from mild to critical [6]. A lot of observational studies have described the clinical characteristics of patients with COVID-19 in Wuhan [7–10], but studies outside Wuhan have rarely been reported. Because of the virus variation, the clinical characteristics of the patients in Wuhan and outside Wuhan maybe different. In this study, we aimed to investigate patients with COVID-19 in Guangzhou to find their clinical characteristics and the risk factors for severe cases. Monitoring these factors can help clinicians identify severe patients early and take subsequent interventions to reduce their illness.

## RESULTS

### Baseline characteristics

Among the 288 patients, 30 cases were in severe group and only 1 case died by the end of the study. Thus, the severity and mortality were 10.4% and 0.3% respectively. The median age of all patients was 48.5 years (IQR 34.3-62), of which women accounted for 54.5% (Table 1). 134 (46.5%) patients had comorbidities, of which cardiovascular disease (CVD) (85, 29.5%) was the most common one, followed by hypertension (84, 29.2%), diabetes (24, 8.3%) (Table 1). 132 patients (45.8%) had a history of exposure to Wuhan 2 weeks before onset (Table 1). The most common symptoms on admission were fever (201, 69.8%) and cough (163, 56.6%), followed by sputum (58, 20.1%), fatigue (43, 14.9%), and myalgia (35, 12.2%) (Table 1).

**Table 1:** Baseline characteristics of non-severe or severe patients of COVID-19 in Guangzhou.

**Demographics and clinical characteristics**	**No. (%)**			**P value**
	**Total (288)**	**Non-severe (258)**	**Severe (30)**	
Age, median (IQR), years	48.5 (34.3-62)	47 (33-61)	61.5(51-71.3)	<0.0001
Age groups (years):	..	..	..	<0.0001
≤30	44(15.3)	44(17.1)	0(0)	..
31-45	87(30.2)	83(32.2)	4(13.3)	..
46-65	116(40.3)	101(39.1)	15(50)	..
≥66	41(14.2)	30(11.6)	11(36.7)	..
Sex:	..	..	..	0.194
Male	131(45.5)	114(44.2)	17(56.7)	..
Female	157(54.5)	114(55.8)	13(43.3)	..
Comorbidity:	..	..	..	..
Hypertension	84(29.2)	69(26.7)	15(50)	0.008
SBP (mm Hg), median (IQR)	125(117-136)	125(117-136)	124.5(117-138.3)	0.186
DBP (mm Hg), median (IQR)	80(74-87)	80(75-87)	80.5(67.3-85)	0.028
MAP (mm Hg), median (IQR)	94.7(87.8-103)	94.7(88-103)	94.8(85.1-102.6)	0.415
Diabetes	24(8.3)	20(7.8)	4(13.3)	0.295
COPD	5(1.7)	3(1.2)	2(6.9)	0.025
CVD	85(29.5)	70(27.1)	15(50)	0.009
Carcinoma	6(2.1)	6(2.3)	0(0)	0.399
CKD	8(2.8)	4(1.6)	4(13.3)	<0.0001
CLD	10(3.5)	8(3.1)	2(6.7)	0.313
Exposure history in Wuhan >2 weeks:	..	..	..	0.016
Yes	132(45.8)	112(43.4)	20(66.7)	..
No	156(54.2)	146(56.6)	10(33.3)	..
Respiratory rate >24 breaths per min	19(6.6)	12(4.7)	7(23.3)	<0.0001
Oxygenation index, median (IQR)	98(97-98.8)	98(97-98.8)	98(97-99)	0.986
Fever (tempetature≥37·3°C)	201(69.8)	174(67.4)	27(90)	0.011
Cough	163(56.6)	142(55)	21(70)	0.118
Sputum	58(20.1)	54(20.9)	4(13.3)	0.326
Myalgia	35(12.2)	30(11.6)	5(16.7)	0.424
Fatigue	43(14.9)	37(14.3)	6(20)	0.410
Nausea or Anorexia	28(9.7)	22(8.5)	6(20)	0.045
Vomiting	6(2.1)	5(1.9)	1(3.3)	0.613
Diarrhea	11(3.8)	6(2.3)	5(16.7)	<0.0001
Headache	26(9)	22(8.5)	4(13.3)	0.385

SBP, Systolic blood pressure; DBP, Diastolic blood pressure; MAP, Mean arterial pressure; COPD, Chronic obstructive pulmonary disease; CVD, Cardiovascular disease; CKD, Chronic kidney disease; CLD, Chronic liver disease.

*P values indicate differences between Severe and Non-severe patients. P<0.05 was considered statistically significant.

### Laboratory and radiological findings

**Figure 1:**
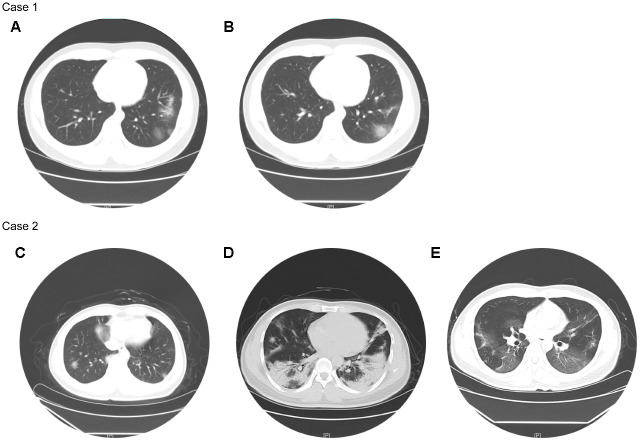
**Chest CTs of two representative cases.** Case 1 (non-severe): Chest CT on Feb 24 (**A**) showed multiple patchy ground-glass opacity in both lungs, with unclear borders and uneven density. Chest CT on Feb 28 (**B**) showed better status, and some lesions were slightly absorbed than before. Case 2 (severe): Chest CT on Jan 29 (**C**) showed the texture of both lungs was slightly increased, and both lungs were scattered in patchy shadows, whose edges were blurred. Chest CT on Feb 11 (**D**) showed the scope of the bilateral lung lesions was enlarged, the density was increased, and the local consolidation and bronchial signs were seen. Chest CT on Mar 4 (**E**) showed improved status, and both lung lesions were significantly less than before.

216 (75%) patients had white blood cells (WBC) in normal range and lymphopenia occurred in 91 (31.6%) patients (Table 2). Compared with non-severe patients, severe patients had significantly reduced serum hemoglobin, platelet and myoglobin, as well as significantly increased WBC, alanine aminotransferase (ALT), aspartate aminotransferase (AST), creatinine, creatine kinase, C-reactive protein (CRP), procalcitonin (PCT), brain natriuretic peptide (BNP), and troponin I (Table 2). 31 (10.8%) patients had unilateral pneumonia, and all of them were non-severe patients; 241 (83.7%) patients had bilateral pneumonia, of which 29 (96.7%) were severe patients (Table 2). Their chest CTs showed varying degrees of patchy ground-glass opacity, with lung lesion area of severe patients usually larger than that of non-severe patients (Figure 1).

**Table 2:** Laboratory and radiological findings of non-severe or severe patients of COVID-19 in Guangzhou.

	**Median (IQR)**			**P value**	**Normal range**
	**Total (288)**	**Non-severe (258)**	**Severe (30)**		
Laboratory findings					
WBC (×10^9^/L)	5.20(4.14-6.44)	5.14(4.10-6.38)	5.33(4.42-7.18)	0.934	4-10
WBC (×10^9^/L) (No (%)):	..	..	..	<0.0001	..
<4	62(21.5)	57(22.1)	5(16.7)	..	..
4-10	216(75)	197(76.4)	19(63.3)	..	..
>10	10(3.5)	4(1.6)	6(20)	..	..
Lymphocyte count (×10^9^/L)	1.42(1.04-1.96)	1.46(1.09-1.97)	1.03(0.84-1.38)	0.511	1.1-3.2
Lymphocyte count (×10^9^/L) (No (%)):	..	..	..	..	..
<1.1	91(31.6)	73(28.3)	18(60)	<0.0001	
Hemoglobin (g/L)	135.5(123-147)	136(125-147.3)	123(114-143.3)	0.001	130-175
Platelet count (×10^9^/L)	194.5(158-247)	199(160.1-249.3)	167(140.3-188.5)	0.043	125-350
D-dimer (mg/L)	1110(700-1700)	1090(680-1600)	1855(865-3442.5)	0.052	<1000
D-dimer (mg/L) (No (%)):	..	..	..	0.106	..
≤1000	125(43.9)	116(45.5)	9(30)	..	..
>1000	160(56.1)	139(54.5)	21(70)	..	..
ALT (U/L)	22.5(14.3-34.5)	22.1(14.2-33.9)	25(16.1-49.1)	0.912	9-50
ALT (U/L) (No (%)):	..	..	..	0.039	..
≤50	254(88.2)	231(89.5)	23(76.7)	..	..
>50	34(11.8)	27(10.5)	7(23.3)	..	..
AST(U/L)	18.4(14.9-25.6)	18.1(14.5-24.5)	21.9(16.8-41.1)	0.161	15-40
AST(U/L) (No (%)):	..	..	..	0.004	..
≤40	256(88.9)	234(90.7)	22(73.3)	..	..
>40	32(11.1)	24(9.3)	8(26.7)	..	..
Myoglobin (μg/L)	15(8.85-22.4)	14.4(8.6-21.2)	27.3(13.1-86.6)	0.212	17.4-105.7
Myoglobin (μg/L) (No (%)):	..	..	..	<0.0001	..
≤106	269(97.1)	247(99.2)	22(78.6)	..	..
>106	8(2.9)	2(0.8)	6(21.4)	..	..
Creatinine (μmol/L)	61.8(50.25-76.56)	62.0(50.4-76.4)	59.6(45.9-78.1)	0.428	54-106
Creatinine (μmol/L) (No (%)):	..	..	..	0.022	..
≤106	279(96.9)	252(97.7)	27(90)	..	..
>106	9(3.1)	6(2.3)	3(10)	..	..
Creatinine kinase (U/L)	52(36-80)	52(37-80)	44.5(27.5-128)	0.238	50-310
Creatinine kinase (U/L) (No (%)):	..	..	..	0.009	..
≤310	283(98.6)	255(99.2)	28(93.3)	..	..
>310	4(1.4)	2(0.8)	2(6.7)	..	..
CRP (mg/L)	9(8-22.72)	9(8-18.9)	24(11.7-51.2)	0.005	<10
CRP (mg/L) (No (%)):	..	..	..	<0.0001	..
≤10	175(60.8)	169(65.5)	6(20)	..	..
>10	113(39.2)	89(34.5)	24(80)	..	..
PCT (ng/mL)	0.13(0.04-32.6)	0.106(0.035-32.58)	0.2(0.09-51)	0.241	<0.05
PCT (ng/mL) (No (%)):	..	..	..	<0.0001	..
<0.05	99(35.5)	96(38.4)	3(10.3)	..	..
0.05-1.0	73(26.2)	56(22.4)	17(58.6)	..	..
1..0-10	6(2.2)	6(2.4)	0(0)	..	..
>10	101(36.2)	92(36.8)	9(31)	..	..
BNP (ng/L)	35(13-117.5)	18.5(9.75-40.25)	213(45-399)	0.014	<100
TNI (μg/L)	0.004(0.001-0.009)	0.003(0.001-0.007)	0.027(0.010-0.099)	0.033	<0.03
TNI (μg/L) (No (%)):	..	..	..	<0.0001	..
≤0.03	168(88.4)	159(92.4)	9(50)	..	..
>0.03	22(11.6)	13(7.6)	9(50)	..	..
Chest radiography findings					
Unilateral pneumonia	31(10.8)	31(12.1)	0(0)	0.044	..
Bilateral pneumonia	241(83.7)	212(82.2)	29(96.7)	0.042	..

WBC, White blood cell; ALT, Alanine transaminase; AST, Aspartate aminotransferase; CRP, C-reactive protein; PCT, Procalcitonin; BNP, Brain natriuretic peptide; TNI, Troponin I.

*P values indicate differences between Severe and Non-severe patients. P<0.05 was considered statistically significant.

### Treatments and outcomes

244 (84.7%) patients received antibiotics, and 233 (80.9%) patients received antiviral drugs (oseltamivir / ribavirin; Table 3). There was a significant difference in the use of glucocorticoids and vasoactive drugs between non-severe and severe patients (Table 3). Five patients were treated with continuous renal replacement therapy (CRRT) and four patients were treated with extracorporeal membrane oxygenation (ECMO), and they were all severe patients (Table 3). 98.9% of the non-severe patients did not take oxygen or took normal-flux oxygen, while 43.3% of the severe patients took high-flux oxygen (Table 3). Eight patients were tracheal intubated and they were all severe patients (Table 3). Severe patients had a significant increase in use of non-invasive mechanical ventilation than non-severe patients (Table 3). Compared with non-severe patients, severe patients were more likely to be transferred to the intensive care unit (ICU), and suffer from ARDS, acute kidney injury and acute cardiac injury (Table 3).

**Table 3:** Treatments and outcomes of non-severe or severe patients of COVID-19 in Guangzhou.

	**No. (%)**			**P value**
	**Total (288)**	**Non-severe (258)**	**Severe (30)**	
Treatments				
Antiviral	233(80.9)	204(79.1)	29(96.7)	0.020
Antibiotics	244(84.7)	214(82.9)	30(100)	0.014
Vasoactive drugs	5(1.7)	1(0.4)	4(13.3)	<0.0001
Glucocorticoid	21(7.3)	12(4.7)	9(30)	<0.0001
CRRT	5(1.7)	0(0)	5(16.7)	<0.0001
ECMO	4(1.4)	0(0)	4(13.3)	<0.0001
Oxygen uptake:	..	..	..	..
None	88(30.6)	84(32.6)	4(13.3)	0.030
Normal-flux	184(63.9)	171(66.3)	13(43.3)	0.013
High-flux	16(5.6)	3(1.2)	13(43.3)	<0.0001
Tracheal intubation	8(2.8)	0(0)	8(26.7)	<0.0001
Tracheotomy	0(0)	0(0)	0(0)	..
Non-invasive mechanical ventilation	32(11.1)	13(5)	19(63.3)	<0.0001
Outcomes				
ICU Admission	27(9.4)	12(4.7)	15(50)	<0.0001
ARDS	3(1)	0(0)	3(10)	<0.0001
Acute kidney injury	5(1.7)	0(0)	5(16.7)	<0.0001
Acute cardiac injury	22(11.6)	13(7.6)	9(50)	<0.0001

CRRT, continuous renal-replacement therapy; ECMO, Extracorporeal membrane oxygenation; ICU, intensive care unit; ARDS, Acute respiratory distress syndrome.

*P values indicate differences between Severe and Non-severe patients. P<0.05 was considered statistically significant.

### Univariate and multivariate analysis of risk factors of severe cases

In univariate logistic regression analysis, we found that older patients with hypertension, chronic kidney disease (CKD), and a history of exposure in Wuhan were more likely to develop severe disease (Table 4). In addition, fever, shortness of breath, diarrhea, WBC, CRP, lymphocytes, COPD, CVD, hemoglobin, ALT, AST, myoglobin, creatinine, creatine kinase, PCT, BNP and TNI were also related with severe cases (Table 4).

**Table 4:** Univariate and multivariate analysis of risk factors of severe cases in Guangzhou.

	**Univariable OR (95% CI)**	**P value**	**Multivariable OR (95%) CI)**	**P value**
Demographics and clinical characteristics				
Age, years	1.063(1.033-1.095)	<0.0001	1.057 (1.018-1.098)	0.004
Female sex (vs male)	0.605(0.282-1.298)	0.197	..	..
Comorbidity present (vs not present)				
Hypertension	2.739(1.272-5.898)	0.010	..	..
COPD	6.296(1.007-39.354)	0.049	..	..
CVD	2.686(1.248-5.780)	0.012	0.986(0.052-18.588)	0.992
CKD	9.769(2.307-41.376)	0.002	6.966(1.310-37.058)	0.023
Respiratory rate >24 breaths per min	6.239(2.238-17.397)	<0.0001	..	..
Exposure history in Wuhan >2 weeks	2.607(1.174-5.791)	0.019	2.765(1.040-7.355)	0.042
Fever (tempetature≥37·3°C)	4.345(1.282-14.730)	0.018	..	..
Nausea or Anorexia	2.682(0.991-7.258)	0.052	..	..
Diarrhea	8.400(2.392-29.494)	0.001	24.349(3.580-165.609)	0.001
Laboratory and radiography findings				
White blood cell count (10^9^/L) (No (%)):	..	..	..	..
≤4	0.910(0.325-2.543)	0.857	0.968(0.289-3.245)	0.958
4-10	1(ref)	..	..	..
≥10	15.553(4.032-59.988)	<0.0001	5.776(1.052-31.722)	0.044
Lymphocyte count (×10^9^/L)	..	..	..	..
<1.1	0.263(0.121-0.573)	0.001	0.697(0.246-1.975)	0.497
Hemoglobin (g/L)	0.966(0.946-0.986)	0.001	..	..
Platelet count (×10^9^/L)	0.993(0.987-1.000)	0.042	..	..
D-dimer (mg/L)	..	..	..	..
≤1000	1(ref)	..	..	..
>1000	1.947(0.859-4.416)	0.111	..	..
ALT (U/L)	..	..	..	..
≤50	1(ref)	..	..	..
>50	2.604(1.022-6.634)	0.045	..	..
AST(U/L)	..	..	..	..
≤40	1(ref)	..	..	..
>40	3.545(1.425-8.823)	0.007	..	..
Myoglobin (μg/L)	..	..	..	..
≤106	1(ref)	..	..	..
>106	33.682(6.413-176.905)	<0.0001	8.910(1.225-64.816)	0.031
Creatinine (μmol/L)	..	..	..	..
≤106	1(ref)	..	..	..
>106	4.667(1.104-19.728)	0.036	..	..
Creatinine kinase (U/L)	..	..	..	..
≤310	1(ref)	..	..	..
>310	9.107(1.234-67.188)	0.030	..	..
CRP (mg/L)	..	..	..	..
≤10	1(ref)	..	..	..
>10	7.596(2.995-19.264)	<0.0001	5.362(1.631-17.626)	0.006
PCT (ng/mL)	..	..	..	..
≤0.05	1(ref)	..	..	..
>0.05	5.333(1.572-18.098)	0.007	..	..
BNP (ng/L)	1.022(1.005-1.040)	0.014	..	..
TNI (μg/L)	..	..	..	..
≤0.03	1(ref)	..	..	..
>0.03	12.231(4.14-36.131)	<0.0001	..	..
Bilateral pneumonia	6.292(0.836-47.378)	0.074	..	..

OR=odds ratio.

*P values indicate differences between Severe and Non-severe patients. P<0.05 was considered statistically significant.

The multivariable logistic regression model was constructed using all variables of significant statistical differences in univariate logistic regression analysis. We found that each 1-year increase in age (OR, 1.057; 95% CI, 1.018-1.098; P=0.004), Wuhan exposure history greater than 2 weeks (OR, 2.765; 95% CI, 1.040-7.355; P=0.042), CKD (OR, 6.966; 95% CI, 1.310-37.058; P = 0.023), diarrhea (OR, 24.349; 95% CI, 3.580-165.609; P=0.001), Myoglobin higher than 106 μg/L (OR, 8.910; 95% CI, 1.225-64.816; P=0.031), WBC higher than 10×10^9^/L (OR, 5.776; 95% CI, 1.052-31.722; P=0.044), and CRP higher than 10 mg/L (OR, 5.362; 95% CI, 1.631-17.626; P=0.006) were independent risk factors for severe cases (Table 4).

## DISCUSSION

Of the 288 patients in our database, only one case (0.3%) died, while the early mortality rate in Wuhan was as high as 28.3% [3]. And the severity of COVID-19 in Guangzhou is 10.4%, which was far less than that in early Wuhan of 31.7% [11].

It was interesting to note Guangzhou patients with Wuhan exposure history had a higher risk of becoming severe cases (Table 4). Earlier reports reported that some patients had SARS-CoV-2 gene fragments missing, suggesting that their virulence gradually weakened [12]. And an article reported that COVID-19 patients in Zhejiang Province had relatively mild symptoms compared with Wuhan [13]. Later, it was reported that SARS-CoV-2 has genomic diversity. It mutated through replication and may evolve under the pressure of immune surveillance in human body, with its virulence, infectivity and transmission being affected [14]. Therefore, the virulence of SARS-CoV-2 may increase or decrease during transmission, and certain populations in different regions may also have a screening effect on it, resulting in different disease degrees and influencing factors of COVID-19 in different regions.

According to previous reports, older age was an important independent predictor of SARS and MERS mortality [15, 16]. Previous studies have confirmed increased severity and mortality of COVID-19 in old patients [3, 7, 17]. A recent study comparing the clinical characteristics and results of COVID-19 patients of different ages showed that the symptoms of elderly patients were more atypical, with more comorbidities, secondary infection, organ injuries, immunodeficiency and a higher risk of critical illness [18]. Many comorbidities in the elderly such as hypertension, diabetes and CKD were treated with ACE inhibitors and angiotensin II receptor blockers, which would upregulate the ACE2 receptor, thereby increasing the risk of SARS-CoV-2 infection and the risk of disease [19]. In our study cohort, age was also one of the risk factors for severe patients (Table 4). Therefore, it’s very important for old patients to have early diagnosis and treat systemic comorbidities carefully.

SARS-CoV-2 was reported to be detected in stool samples from patients [20], and a study of a family cluster have reported two COVID-19 patients who had only diarrhea symptom [9]. Besides diarrhea, some patients also had other gastrointestinal symptoms such as vomiting and abdominal pain [21]. Our analysis showed that diarrhea was a risk factor for severe cases (Table 4), which suggested that beside of damaging the respiratory system, the virus may also have a certain function on the digestive system. This finding may be related to the expression of SARS-CoV-2 receptor ACE2 in both the epithelial cells of lungs and digestive tract [21, 22]. Given the small number of diarrhea cases (11, 3.8%) (Table 1), SARS-CoV-2-induced digestive system damage may also be related to other physical factors of these patients, which deserves further study.

Previous studies have reported that COVID-19 non-survivors had more neutrophil counts than survivors, which may be related to cytokine storms caused by virus invasion [7, 11]. Our analysis found that elevated WBC and CRP were risk factors for severe cases (Table 4). Like neutrophils, WBC and CRP are also indicators of inflammatory status in the body. When they elevated, there may be a cytokine storm caused by virus invasion in the body, which may cause severe inflammation in lungs and other organs, and aggravate the disease. Therefore, paying close attention to changes in WBC, CRP and making timely correction can effectively reduce the number of severe cases and deaths.

In our study, myoglobin, creatine kinase, BNP, and TNI were increased in severe patients compared to non-severe patients (Table 2), and myoglobin was a risk factor for severe patients, which indicated that COVID-19 may be related to acute cardiac injury. ACE2 is also expressed in heart [23], and SARS-CoV has been shown in animal models to directly mediate myocardial inflammation and damage by down-regulating myocardial ACE2 and lead to poor cardiac prognosis [24]. A meta-analysis involving 4189 patients showed that more severe COVID-19 was associated with increased troponin, creatine kinase, myoglobin, and NT-proBNP [25]. Myoglobin was also included in the COVID-19 severity score table as one of the biomarkers [26]. The severity of COVID-19 may be related to acute cardiac injury, which prompts us to effectively monitor heart condition to prevent COVID-19 patients from myocarditis and avoid poor cardiac prognosis.

Many studies have reported that comorbidities were major risk factors for increasing COVID mortality and poor prognosis [7, 8], and CKD was one of them. Due to older age, previous comorbidities, impaired immune system, and regular visits to crowded outpatient dialysis centers, CKD patients have increase susceptibility to SARS-COV-2 [27]. On one hand, the above factors have greatly reduced the ability of CKD patients to overcome the virus and may lead to severe disease or even death. On the other hand, SARS-COV-2 can directly damage kidney by combine with ACE2 [28], and cause kidney inflammation and acute kidney injury [13, 29], which was consistent with the increase creatinine level of severe patients in our study. AKI could further aggravate CKD as well as worsening the patients’ whole conditions, leading patients to develop severe illness.

The study has several limitations. First, the sample size of our study cohort was relatively small including only 288 patients from a single center. Due to the exploratory nature of the study, which was not driven by formal hypotheses, we did not estimate the sample size, but included as many cases as possible. Second, this study lacked laboratory data such as serum cytokines and chemokines, so that we cannot evaluate the inflammation levels and cytokine storms of these patients. Third, this was a retrospective study. The data in this study was only a preliminary assessment of clinical characteristics and risk factors of COVID-19 severe patients. Further researches are still needed.

In conclusion, our research showed that the severity and mortality of COVID-19 in Guangzhou were much lower than those in early Wuhan. The risk factors for severe cases of COVID-19 in Guangzhou included older age, Wuhan exposure history greater than 2 weeks, diarrhea, elevated Myoglobin, elevated WBC and CRP, and CKD. Investigating and monitoring these factors can help clinicians identify patients with poor prognosis at an early stage, and take proactive interventions to benefit patients and reduce severity and mortality. It also provided significant experience and reference for countries around the world to fight against COVID-19.

## MATERIALS AND METHODS

### Study design and participants

This single-center, retrospective cohort study was conducted at Guangzhou Eighth People’s Hospital (Guangzhou, China), which was the designated hospital to treat patients with COVID-19 in Guangzhou. From Jan 15, 2020 to Mar 10, 2020, we recruited 288 adult patients with COVID-19 (the total number was 292, including 4 underage patients).

This study was approved by the Ethics Committee of Guangzhou Eighth People’s Hospital, and informed consent was obtained from all patients enrolled.

### Definitions

According to the Chinese diagnosis and treatment guideline for COVID-19 (trial version 7.0) [6], 288 patients were divided into non-severe group (258 cases), including light and general patients, and severe group (30 cases), including severe and critical patients. A case was defined as severe if it met any of the following: (1) shortness of breath, respiratory rate ≥ 30 times / minute; (2) blood oxygen saturation ≤ 93% at rest; (3) oxygenation index (PaO2 / FiO2) ≤ 300 mmHg; (4) pulmonary infiltrates > 50% of the lung lesions within 24-48 hours; (5) respiratory failure, requiring mechanical ventilation; (6) shock; (7) combine with multiple organ dysfunction, needing ICU monitoring treatment. Acute Respiratory Distress Syndrome (ARDS) was defined according to WHO’s guidance for COVID-19 [30]. Acute renal injury (ARI) was determined from serum creatinine [31]. Acute cardiac injury (ACI) was determined based on the serum concentration of troponin I (TNI) [11]. The reference ranges of all laboratory inspection indicators were measured in the laboratory of Guangzhou Eighth People’s Hospital.

### Data collection

This study reviewed the clinical electronic medical records, nursing records, laboratory tests and radiological findings of 288 adult patients with COVID-19, who were confirmed by nucleic acid testing. And we extracted epidemiology, demographics, clinical manifestations, laboratory data, chest radiography findings, treatment and outcome data for statistical analysis and research.

### Statistical analysis

The purpose of this study was to analyze the risk factors of severe patients by comparing severe group and non-severe group in terms of their clinical data. Therefore, no formal assumptions were used to facilitate the calculation of the sample size, and we included the largest number of patients who met the inclusion criteria.

We represented continuous variables as median and interquartile range (IQR), and categorical variables as frequency (N) and percentage (%). We assessed differences between severe group and non-severe group using two-sample *t* test or the Mann-Whitney U test depending on parametric or nonparametric data for continuous variables, and χ 2 test or Fisher’s exact test for categorical variables. Univariate and multivariate logistic regression models were used to explore the risk factors for severe cases.

A P value of less than 0.05 was considered statistically significant. All data were statistically analyzed using SPSS software (version 25).
